# The Optimization of Pressure-Assisted Microsyringe (PAM) 3D Printing Parameters for the Development of Sustainable Starch-Based Patches

**DOI:** 10.3390/polym15183792

**Published:** 2023-09-17

**Authors:** Carmen Laura Pérez Gutiérrez, Francesco Cottone, Cinzia Pagano, Alessandro Di Michele, Debora Puglia, Francesca Luzi, Franco Dominici, Rossella Sinisi, Maurizio Ricci, César Antonio Viseras Iborra, Luana Perioli

**Affiliations:** 1 Department of Pharmaceutical Sciences, University of Perugia, 06123 Perugia, Italy; carmenlaura.perezgutierrez@studenti.unipg.it (C.L.P.G.); rossellasinisi97@gmail.com (R.S.); maurizio.ricci@unipg.it (M.R.); luana.perioli@unipg.it (L.P.); 2 Department of Pharmacy and Pharmaceutical Technology, Faculty of Pharmacy, University of Granada, 18071 Granada, Spain; cviseras@ugr.es; 3 Department of Physics and Geology, University of Perugia, 06123 Perugia, Italy; alessandro.dimichele@unipg.it; 4 Civil and Environmental Engineering Department, University of Perugia, UdR INSTM, 05100 Terni, Italy; debora.puglia@unipg.it (D.P.); franco.dominici@unipg.it (F.D.); 5 Department of Materials, Environmental Sciences and Urban Planning (SIMAU), 60131 Ancona, Italy; f.luzi@staff.univpm.it

**Keywords:** 3D printing, extrusion-based technique, PAM, starch gel, β-glucan, patch

## Abstract

The aim of this work was to develop sustainable patches for wound application, using the biopolymer starch, created using a low-cost 3D printing PAM device. The composition of a starch gel was optimized for PAM extrusion: corn starch 10% *w*/*w*, β-glucan water suspension (filler, 1% *w*/*w*), glycerol (plasticizer, 29% *w*/*w*), and water 60% *w*/*w*. The most suitable 3D printing parameters were optimized as well (nozzle size 0.8 mm, layer height 0.2 mm, infill 100%, volumetric flow rate 3.02 mm^3^/s, and print speed 15 mm/s). The suitable conditions for post-printing drying were set at 37 °C for 24 h. The obtained patch was homogenous but with low mechanical resistance. To solve this problem, the starch gel was extruded over an alginate support, which, after drying, becomes an integral part of the product, constituting the backing layer of the final formulation. This approach significantly improved the physicochemical and post-printing properties of the final bilayer patch, showing suitable mechanical properties such as elastic modulus (3.80 ± 0.82 MPa), strength (0.92 ± 0.08 MPa), and deformation at break (50 ± 1%). The obtained results suggest the possibility of low-cost production of patches for wound treatment by additive manufacturing technology.

## 1. Introduction

In recent years, the need for patient-tailored treatments with a focus on environmental care has grown exponentially. From this point of view, the use of 3D printing represents a tool that has recently assumed importance. Three-dimensional printing, in fact, is an innovative technology able to both produce customized formulations, which overcome the limitations of traditional medical devices and medicines, and respect the environment [1,2,3,4,5].

Three-dimensional printing is an additive manufacturing (AM) technology based on the principle of layer-by-layer deposition of a material to fabricate a desired 3D object [6] that is designed beforehand with computer-aided design (CAD) software. Three-dimensional printing is also a scalable and sustainable technique used in many fields [7] and is considered very useful for preparing ad hoc formulations developed to satisfy specific patient needs. In the pharmaceutical field, 3D printing can be employed for the fabrication of a wide assortment of medical devices and dosage forms varying in shape, release profile, and drug combination (e.g., polypills, orodispersible film, microneedles, transdermal patches, etc.) [8,9].

A generally accepted classification of the different 3D printing methods for both pharmaceutical and medical applications is based on three main groups.

(i) Ink-jet printing-based techniques such as continuous ink-jet printing (CIJ) and drop-on-demand printing DOD [10]; (ii) laser-based writing such as stereolithography (SLA), selective laser sintering (SLS), and Digital Micromirror Device (DMD); (iii) nozzle-based deposition as fused deposition modeling (FDM) and PAM methods [11].

Nozzle-based 3D printing technologies were extensively studied and also attracted the greatest interest due to their versatility, reproducibility, and high scalability potential. Between these, the PAM technique is the most interesting and suitable for application in both pharmaceutical and medical fields. This technique, also known as semisolid extrusion (SSE), is based on the extrusion of a semisolid formulation (e.g., hydrogel, pastes) and requires a following drying treatment of the extruded product in order to obtain the final product. The printing processes can be carried out at room temperature (R.T.), resulting in suitable thermosensitive materials (both excipients and drugs). Generally, the semisolids are prepared using synthetic and semi-synthetic polymers such as hydroxypropyl methylcellulose (HPMC) and polyvinylpyrrolidone (PVP), microcrystalline cellulose (MCC) [12], and suitable and non-toxic solvents like water and ethanol.

On 30 August 2022, the European Committee (EC) released a draft proposal to restrict the use of intentionally added microplastics to prevent the release of 500,000 tons of microplastics into the environment over a 20-year period [13]. Microplastics, in fact, represent a serious problem for humans, as every day, people are exposed to those present in health care products, foods, and in the environment with a consequent increased incidence of many pathologies [14]. According to EC restrictions, synthetic/semi-synthetic polymers, generally used in health products, could be classified as microplastics, suggesting the necessity to find suitable alternatives from natural sources to be employed in the preparation of medicines, medical devices, cosmetics, and all health care products.

In this perspective, the development of patches by means of the PAM technique based on the biopolymer starch was investigated and demonstrated in this work.

Starch is a natural biodegradable polysaccharide, classified as G.R.A.S. (generally recognized as safe) by the FDA due to its safety and biocompatibility. It is already used in the pharmaceutical industry as an excipient, especially in the preparation of pharmaceutical dosage forms for oral use (powders, tablets, capsules); dermatological use (hydrogels, pastes); and powders for external use (talcs) [15,16]. Starch consists of a soluble (amylose) and insoluble (amylopectin) fraction and shows different behaviors as a function of the temperature when dispersed in water. Starch granules are insoluble in cold water because of their high crystallinity. When they are dispersed in hot water (~80 °C), below their glass transition temperature, they swell, the crystalline structure breaks down, and water molecules bind through hydrogen bonds to the carboxyl groups of amylose and amylopectin forming a tridimensional structure responsible for water gelification [17]. The gel obtained from starch is not suitable for extrusion; for this reason, an optimization of the composition is necessary.

The aim of this work was to optimize and develop a starch-based gel suitable for PAM extrusion by a customized 3D printer in order to prepare a non-toxic and bio-sustainable patch suitable for wound treatment. The optimization of the most suitable 3D printing settings was investigated as well.

This topic represents an unexplored field because data in the literature only show the use of starch gel for patch production by casting method [18,19] and electrospinning [20,21]. The performance of the starch gel was improved by combining it with a plasticizing agent (glycerol) and a filler. About the latter, considering the promising results obtained in a previous work [22], it was used a water suspension of β-glucan obtained from barley flour by high-power ultrasonic (HPU) technique. There are no other studies in the literature dealing with the purpose of the starch gel composition studied, as well as its use for PAM 3D printing and the setting of the specific parameters for this formulation. Thus, this topic is worthy of investigation from the perspective purpose of new safe and environmentally friendly health products by the scalable technique of PAM 3D printing.

## 2. Materials and Methods

### 2.1. Materials

Corn starch (CS) and glycerol (Gly) were supplied by A.C.E.F. s.p.a. (Fiorenzuola d’Arda, Italy). Alginic acid sodium salt from algae (marine), MW 216.12, M/G (mannuronic acid/guluronic acid) (ratio = 1.56; viscosity 1% in water 15–25 cps), was acquired from Sigma-Aldrich (Milano, Italy). Barley seeds (cultivated in Monteleone di Spoleto, Perugia, Italy) were purchased from Antica Spezieria Bavicchi (Perugia, Italy). Ultrapure water was obtained by reserve osmosis process in a MilliQ system Millipore (Rome, Italy). Other reagents and solvents were of analytical grade and used without further purification.

The simulated wound fluid (SWF), pH 6.5, was prepared by dissolving 8.30 g of NaCl and 0.28 g of CaCl_2_ in 1000 mL of ultrapure water.

### 2.2. CS Gels Preparation

CS gel was prepared using the composition of starch glycerolate reported in the corresponding monograph of Farmacopea Ufficiale Italiana (F.U. XII Ed) and properly modified. The modification consisted of the addition of a filler in order to improve the resistance of the final xerogel.

The final CS gel composition is the follows:

CS 10% *w*/*w;*
Gly 29% *w*/*w;*
Diller 1% *w*/*w;*
Distilled water 60% *w*/*w.*

The CS gel was prepared following the procedure described by Valencia et al. and Perotti et al. and was suitably modified [23,24].

The filler 1% *w*/*w*, represented by a water suspension of βglucan (βglu), was dispersed in distilled water 60% *w*/*w* under magnetic stirring (600 rpm) for 30 min at R.T. Then, Gly 29% *w*/*w* was added under magnetic stirring (600 rpm) for 15 min at R.T. The dispersion was warmed at 80 °C, and then, CS 10% *w*/*w* was added under magnetic stirring (600 rpm) and left to stir at 80 °C until gelification (~7 min). The obtained gel was then put into a cone syringe sterile single-use 60-catheter suitable for PAM extrusion and left to cool at room temperature (R.T) for 24 h. During this period, the air bubbles incorporated during the gel preparation are completely removed as they spontaneously leave the formulation.

βglu suspension (βglucan content 6.30 ± 0.70 g/100 g) was prepared according to a previous work [22]. Briefly, barley grains were ground by the knife mill GRINDOMIX GM 200 (Retsch, Predengo, Cremona, Italy) working at 4000 rpm for 3.30 min. The obtained flour was sieved by stainless steel sieves (Endecotts Ltd., London, UK) in order to select the fraction with a size range of 400–500 μm. According to reference [25], this selected fraction was boiled in EtOH (80%, *v*/*v*) under reflux for 2 h in order to inactivate β-gluconases enzymes responsible for βglu enzymatic hydrolysis. Thereafter, the suspension was centrifuged (Hettich zentrifugen, Universal 32R) for 10 min at 4000 rpm, 20 °C, to remove EtOH. A total of 150 mL of water was added to the solid and was treated by a high-power ultrasonic (HPU) technique working in the following conditions: emitted power of 750 W and transmitted power of 200 W, frequency of 20 KHz, amplitude of 50% working at 55 °C, for 15 min, using Horn 330 Type ultrasonic probe VCX750 (SONICS, Newtown, CT, USA). Then, the obtained suspension was centrifuged (10 min, 4000 rpm, 20 °C) and the supernatant was collected. The solid was re-dispersed in 150 mL of distilled water to repeat the HPU treatment for other 2 cycles. A total of 3 cycles were performed.

### 2.3. Design and Printing Parameters of Customized Patches

**Figure 1:**
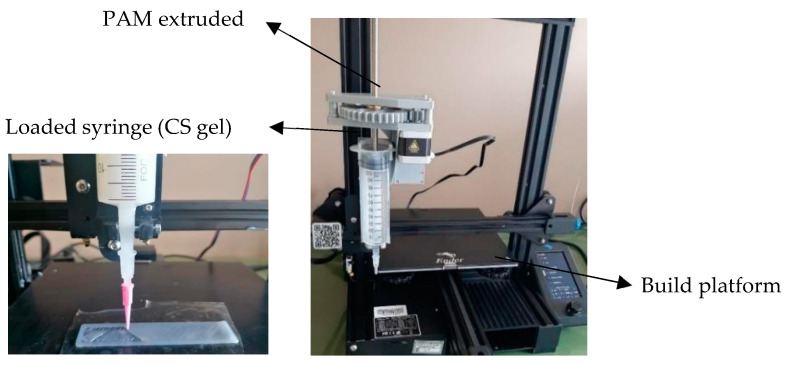
Creality 3D Ender-3 V2 modified for 3D printing by PAM technique.

Rectangular patches having 10 cm × 2 cm dimensions were designed by CAD software (OnShape education) and exported as .stl file. The .stl file was then imported into a slicer software (Ultimaker Cura 4.0) in which printing parameters, such as the layer height, print speed, infill percentage, and flow rate, were selected (Table 1) in order to optimize the extrusion-based 3D printing. Patches were prepared by extrusion-based 3D printing technique PAM by means of a customidez Creality 3D Ender-3 V2 (Shenzhen Creality 3D technology Co., China) that was equipped with syringe extruder (Figure 1).

The gels were extruded and deposited by printing on different support materials, namely Teflon (PTFE), plastic (PET), and silicone. The wet patches were then dried in oven at different temperatures (37 °C, 40 °C, and 70 °C) and for different times (12 h, 16 h, and 24 h) in order to select the best post-processing conditions. All the dried patches were then conditioned at R.T. under two different relative humidity (RH) conditions: 40% RH under CaCl_2_ and 37% RH under SiO_2._

#### 2.3.1. Nozzle Diameter

The selection of the most suitable nozzle diameter (syringe tip) was performed on the basis of (i) gel consistency, (ii) gel rheological behavior, and (iii) quality of the printed material [26]. Different nozzle diameters, from 0.80 mm to 1.62 mm, were evaluated to extrude the material.

#### 2.3.2. Infill Density

The infill consists of the internal structure of the three-dimensional object, which can have different patterns and shapes; the infill density is a percentage value of the volume of material present in each layer that fills the inner part of the print. If the infill is 100%, the internal structure is full, while setting the infill to 0% will result in the object being empty.

#### 2.3.3. Flow Rate

This parameter is a parameter you enter into the slicing software to control filament flow and tells the printer how much of the filament’s cross-sectional area should be extruded per unit time. In the slicer software, it is expressed as a percentage value relative to the full extrusion rate, and it has been optimized by ranging from 30% to 80%. From this value, it is possible to retrieve the exact volumetric flow rate as described in Equation (1). A low flux value allows for obtaining an object with homogeneous surface and low resolution, while high values can lead to the formation of surfaces with low resolution.

#### 2.3.4. Volumetric Flow Rate

The volumetric flow rate through the printer nozzle is calculated from the pre-set print speed, that is, the speed at which molten material is deposited from the syringe nozzle to the bed. The volume flow rate, *Q,* can be calculated using the radius of the nozzle, *r*, and the printing speed, *v*:

(1) $$Q=\pi r^{2}v{(f}_{r}/100)$$

#### 2.3.5. Print Speed

The printing speed represents the movement of the extruder with respect to time (distance/time) and can be expressed as mm/s. Different print speeds were evaluated in a range from 10 mm/s to 30 mm/s, and the selection of the set value was made considering the extrudability of the gel. The printing speed is also adjusted on the basis of the characteristics of the printed layer.

#### 2.3.6. Material Extrusion Rate

The material extrusion rate is the amount of material extruded at different printing speeds when a specific pressure is applied through a nozzle of fixed dimension; it is expressed in mg/min. In order to design and develop customized suitable patch, the optimization of the material extrusion rate is required; it determines the amount of extruded material in unit time.

### 2.4. Rheological Measurements

Viscometry analysis and rheometric Scientific’s Ares model were carried out by a rotational rheometer with parallel flat plate geometry (diameter 25 mm). Mode: Dynamic Rate Sweep Test (RST), Transient Step Rate Test (SRT), and Dynamic Temperature Step Test (DTSt). The experiments were performed in triplicate; each result represents the average of the three measurements.

### 2.5. Optimization of Post-Printing Process

The 3D printing of the extruded gel was carried out on different support materials, including polytetrafluoroethylene (PTFE), polyethylene terephthalate (PET), and silicone, to observe the impact of the support on (i) the material spreadability, (ii) the integrity (iii) and the homogeneity of the final product. The support for printing is also important to determine post-printing process conditions such as temperature and drying time. In order to evaluate the most suitable final structure, monolayer patches (0.2 mm total thickness), bi-layered patches (0.4 mm total thickness), and three-layered patches (0.6 mm total thickness) were prepared.

### 2.6. Backing Layer Preparation

The backing layer was prepared by casting method using an Alg gel at 1.5% *w*/*w*, according to a previous work [27], and had the following composition: Alg 1.5% *w*/*w*, Gly 10% *w*/*w*, distilled water 88.5% *w*/*w*. The preparation of this backing layer was carried out under magnetic stirring (600 rpm) for 90 min. Alg gel (385 mL) was cast in plastic supports (24 × 34 cm) and dried in oven at 37 °C for 50 h. Afterwards, it was immersed in a solution of CaCl_2_ at 5% *w*/*v* for 3 min in order to promote Alg polymerization and dried in oven at 37 °C for 24 h. Then, the obtained film was washed with distilled water to remove the excess of CaCl_2_ and finally dried at 37 °C for 24 h.

### 2.7. Patches Characterization

#### 2.7.1. Scanning Electron Microscopy (SEM)

The morphology and thickness of the prepared patches were investigated by Scanning Electron Microscopy using an FE-SEM LEO 1525 ZEISS (Carl Zeiss Microscopy, Jena, Germany). The sample was deposited on conductive carbon adhesive tape, and metallization with chromium (8 nm) by sputtering was carried out. The images were acquired by means of In-lens detector with an electron high tension (EHT) of 5 kV.

#### 2.7.2. Fourier-Transform Infrared Spectroscopy (FT-IR)

Infrared (IR) spectra of the samples and all their components were registered by a Shimadzu IR Spirit QATRS spectrometer (Kyoto, Japan).

#### 2.7.3. Mechanical Characterization

Tensile tests were performed by using a digital microprocessor instrument (Llyod LR30K, Lloyd Instrument, Fareham, UK). The patches were cut in portions 100 mm × 10 mm [28] (UNI ISO 527) to have a useful length of 50 mm. The experiment was performed at 5 mm/min, cell load 50 N. The two ends of the patch were fixed with clamps to the dynamometer. The sample was subjected to tensile stress until deformation and break. Values for maximum stress, deformation at maximum stress, and elastic modulus were registered. The obtained results are an average of five measurements (*n* = 5). The formulation was stored at R.T. for 1 week under vacuum conditions and in oversaturated calcium chloride (CaCl_2_) solution.

#### 2.7.4. Ex Vivo Adhesion Studies

**Figure 2:**
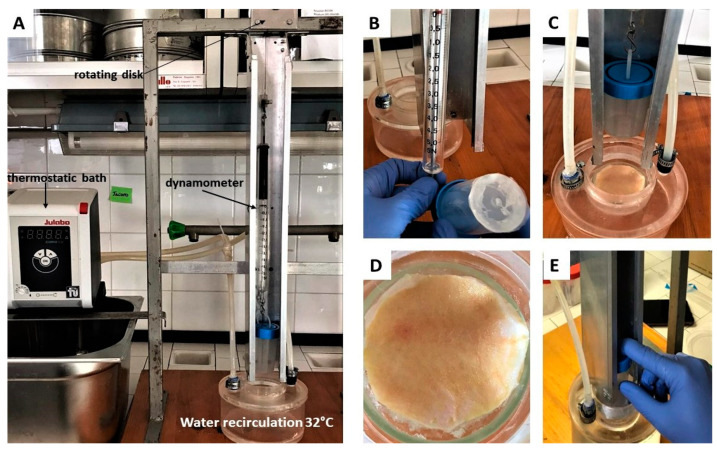
(**A**) Experimental setup used to perform the ex vivo adhesion studies; (**B**) dynamometer to which a support (at the bottom of which the patch is then fixed) is connected; (**C**) porcine skin tissue fixed on the surface of glass support, thermostat of 32.0 °C ± 0.5; (**D**) porcine skin circle (diameter 4 cm); (**E**) contact between patch (on the bottom of the support) and skin.

Patches adhesion force was assessed using pig skin samples (from shoulder region) obtained from Large White pigs weighing ~165–175 kg, supplied by Veterinary Service of ASL N. 1 Città di Castello (Perugia, Italy) and used within 12 h of pig death. The ex vivo adhesion force was measured by a dynamometer (Didatronic, Treni, Italy) (Figure 2A). The patch was attached to a support using cyanoacrylate glue and connected to the dynamometer Didatronic (Whatman GmbH, Dassel, Germany), as shown in Figure 2B. A piece of porcine skin tissue was fixed with cyanoacrylate glue on the surface of glass support and placed in a thermostatic bath at 32.0 °C ± 0.5 (Figure 2C). The portion of pig skin was cut to have a precise diameter of 4 cm (Figure 2D). The free side of the patch was wetted with 150 µL of SWF and put in contact with the skin sample by applying a light force for 2 min (Figure 2E). The force necessary for patch detachment from the skin was measured and expressed as an average of three measurements (*n* = 3) [27].

## 3. Results and Discussion

### 3.1. Optimization of Gel Formulation

CS is an abundant natural source and is already used in health products for many applications as it is safe and classified as G.R.A.S. (generally recognized as safe) by the FDA. Furthermore, it is also cheap, biodegradable, and biocompatible. For these reasons, this raw material was considered interesting as a potential polymer for the development of sustainable patches by 3D printing PAM technique.

In the Farmacopea Ufficiale Italiana F.U. XII Ed., there are monographs of many starch-based formulations for dermatological use. The starting point of this study was the recipe of starch glycerolate consisting of corn starch (CS) 10% *w*/*w*, glycerol (Gly) 70% *w*/*w,* and bidistilled water (W) 20% *w*/*w*.

However, this gel is not suitable for PAM extrusion as it does not allow a regular flow through the syringe nozzle. For this reason, some modifications of the composition were carried out, and six gels were prepared, as reported in Table 2. CS % was fixed at 10% *w*/*w* while Gly content was reduced.

Firstly, the extrusion ability through the syringe nozzle was evaluated for the prepared gels (Table 2). G1 and G2 were very viscous (responsible for nozzle clogging), while G6 was too liquid (leakage from the syringe); for these reasons, these gels were considered not suitable for printing and thus excluded.

The remaining formulations, namely G3, G4, and G5 gels, were more suitable for the purpose and were further characterized. A preliminary printing attitude evaluation showed that these three gels did not allow for the obtaining of a homogeneous object. During the extrusion, in fact, the gel fragmentation occurred with consequent imperfections in the final object. Thus, a further modification of the compositions of gels G3, G4, and G5 was made by introducing a filler from a natural source [29] (Table 3). In a previous work [22], the suitability of a βglucan (βglu) water suspension as filler for polymeric patches was assessed. For this reason, it was decided to use it. Considering that the amount of filler generally useful to improve the mechanical properties of polymeric films is 1% *w*/*w* [30], this percentage of βglu was introduced in the formulation composition. The obtained gels (Table 3) were submitted for a preliminary evaluation of the extrusion ability.

The product printed using both G3-βglu gel and G5-βglu gel did not allow for obtaining uniform objects showing the desired shape (as designed from CAD). This is attributable to the consistency of the two gels. G3-βglu gel is probably too viscous due to the low water content (50% *w*/*w*), while G5-βglu gel is too fluid due to the high water content (70% *w*/*w*). In both cases, the consistency was not suitable, and for this reason, G3-βglu and G5-βglu gels were excluded from the study. In the case of G4-βglu gel, it was possible to obtain an object able to maintain the features set by CAD and, for this reason, considered useful for the achievement of fixed objectives. Then, the selected gel was deeply characterized, and in the first instance, the rheological properties were evaluated. The estimation of gel viscosity has a significant impact on the quality of the final printed object [31]. Generally, for PAM printing, it is preferable to use semisolid formulations having shear-thinning behavior, as this property is responsible for the ability to be pushed through a nozzle [3,32].

**Figure 3:**
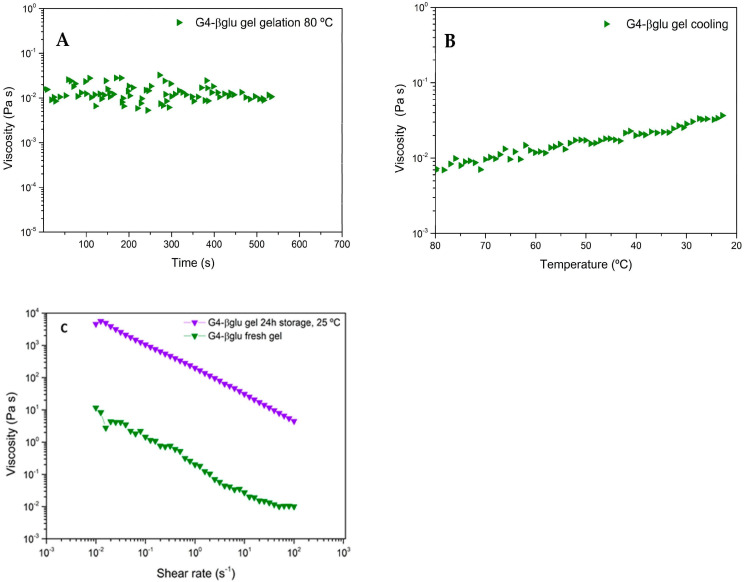
Rheograms of G4-βglu gel (**A**) viscosity (Pa·s) vs. time (s^−1^) showing the gel formation at 80 °C; (**B**) viscosity (Pa·s) vs. temperature (°C); (**C**) viscosity (Pa·s) vs. shear rate (s^−1^) of the gel just after preparation and after storage at 25 °C for 24 h; (*n* = 3).

The G4-βglu gel formation process at 80 °C was evaluated by rheological analysis by SRT, and the obtained rheogram (Figure 3A) shows that until 420 s (~7 min), the viscosity values change in a wide range; then, at 420 s, the trend line of viscosity as a function of time begins to be constant, indicative of gel formation. The gel cooling process was studied by DTSt, and as shown in Figure 3B, the viscosity increases as temperature decreases as a consequence of gel consistency improvement during the cooling.

As gel consistency varies as a function of the temperature, it was very important to evaluate its viscosity after preparation and after storage at a fixed time (24 h) and temperature (25 °C). As shown in Figure 3C, the viscosity measured by RST analysis for the gel after preparation, called G4-βglu fresh gel, is approximately in the range between 10 ^−2^ and 10 Pa·s in the shear rate range 10^1^–10^−1^ s^−1^ and is not linear. This is ascribable to the necessity of a time of stabilization for the newly formed gel. In fact, the viscosity measured for the same gel stored at 25 °C for 24 h is almost linear (Figure 3C), and the shear rate range 10^1^–10^−1^ s^−1^ resulted in improvements (10^1^ and 10^3^ Pa·s), demonstrating the stabilization of the polymeric network. The decrease in viscosity as a consequence of shear rate increase is indicative of shear-thinning behavior, in which an increase in shear rates leads to polymeric chain alignment and an ordered structure. Increased shear rate imitates the extrusion of gel via the nozzle, where viscosity reduction results in uniform extrusion [33].

### 3.2. Design and Printing Parameters of Customized Patches

#### 3.2.1. Nozzle Diameter

**Figure 4:**
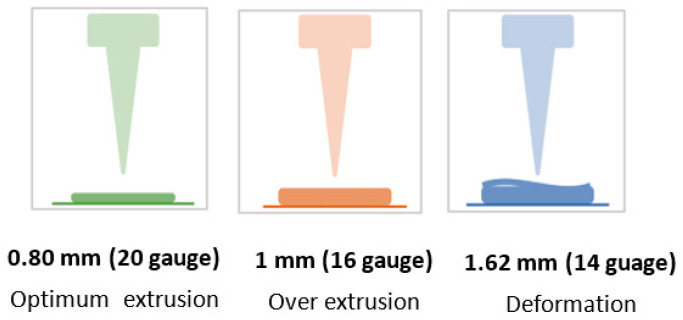
Schematic representation of behavior during extrusion using tips with different internal diameters.

The first parameter considered is the nozzle diameter. This parameter has a considerable influence on the precision of the printing process adhesion between layers and can also influence the gel extrusion [34]. Three nozzle diameters, 0.8 mm, 1 mm, and 1.62 mm, were investigated (Figure 4). The use of a 0.80 mm nozzle produced a line of uniform thickness, resulting in an optimal extrusion. Using a 1 mm nozzle, the deposition line was continuous, but the layer thickness was larger than the nozzle size, resulting in an excessive amount of extruded material; the use of a 1.62 mm nozzle caused the deformation of the printed layer and was thus considered inadequate. Thus, a diameter nozzle of 0.80 mm was selected.

#### 3.2.2. Infill Density

All tests were performed with the filler fixed at 100%. This could increase the strength and stiffness of the printed object and thus improve the mechanical characteristics of the planned structure.

#### 3.2.3. Flow Rate

The flow rate value indicates the speed at which the printer extrudes a given amount of material; this parameter influences many other factors, such as the speed at which the extruder motor moves (step/min), under- or over-extrusion of material, adhesion to the substrate, and line width. This parameter is selected in the slicing software and is expressed in percentage as it is normalized with respect to the full extrusion rate of the printer. The corresponding volumetric flow rate (VFR) is calculated by Equation (1). The behavior of the gel during extrusion and the amount of deposited material were evaluated in a range between 30% and 80% with a printing speed of 15 mm/s. The choice to investigate this range comes from preliminary practical considerations; in fact, for flow rate below 30%, the printing did not happen, while for values > 80%, the extrusion was very fast, and the printing was not accurate. As shown in Table 4, low flow values resulted in better resolution, higher uniformity in material deposition, and lower deformation than high flow rates. After evaluating the quality of the printed patches in terms of resolution, presence of imperfections, and material deposition, a flow rate value of 40% was selected (Patch F_40).

#### 3.2.4. Print Speed

Starting from Patch F_40, the print speed was evaluated.

The print speed defines the speed, expressed as mm/s, at which the extruder (syringe) moves along the X and Y axes across the printing plate during the printing process.

A print speed range of 10–30 mm/s was evaluated (Table 5). Using high print speed values (20–30 mm/s), the prints suffered from poor quality and lower accuracy; at lower values (10–15 mm/s), a more homogeneous extrusion of the material and higher resolution of the printed patch structure was observed. However, using the print speed of 10 mm/s, an uneven distribution of the material was observed due to the noticeably slower movement of the extruder, resulting in significant variations in the thickness of the extruded gel. By applying a print speed of 15 mm/s, it was possible to obtain a continuous extrusion, obtaining a uniform patch (Patch F_40_S_15) and resulting in a suitable parameter.

#### 3.2.5. Material Extrusion Rate

The material extrusion rate is a parameter that determines the amount of material extruded per unit of time. This parameter was determined at different printing speeds, with the flow rate fixed at 40%. The patches evaluated were F_40_S_10, F_40_S_15, F_40_S_20, F_40_S_25, and F_40_S_30.

**Figure 5:**
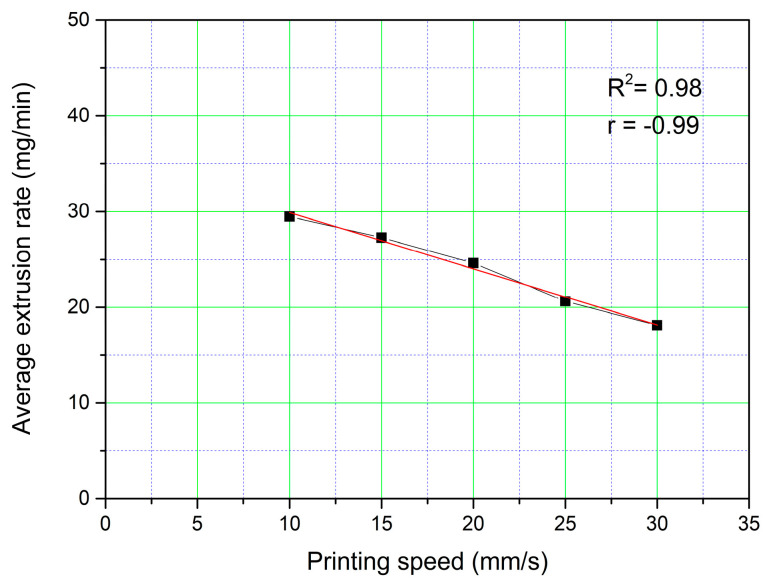
Effect of print speed on the extrusion rate of the material.

A linear decrease (r = −0.99) of the amount of extruded material in one minute can be observed with respect to the increase in the printing speed. The coefficient of determination (R^2^) value of 0.98 is shown in Figure 5.

### 3.3. Optimization of Post-Printing Process

An important aspect to consider when the PAM technique is used is the selection of the most suitable platform on which the process of extrusion takes place. In fact, this can be constituted by different materials, and, generally, the one that is not capable of establishing interactions with the extruded semisolid must be selected. In this study, three materials were selected: PTFE, PET, and silicone. Moreover, the printed object requires a post-printing drying process in order to remove the solvent (water) and to obtain the final planned object. The drying process is, therefore, crucial to obtain resistant patches that are elastic and intact. For an optimal drying process, both temperature and time must be carefully set; too high temperatures or too long times may lead to excessive solvent evaporation, resulting in patch deformation and shrinking. On the other hand, low temperatures and short heat exposure times lead to inadequate water removal, resulting in sticky patches easily susceptible to microbiological contamination. Using G4-βglu gel, the patches were printed on three different selected supports: PTFE, PET, and silicone. For each support, three samples were printed and submitted to different drying conditions in an oven, as reported in Table 6. By this study, it was possible to select the most suitable drying conditions as well as the most suitable support for printing. From the observation of the final printed objects (patches), it emerged that the silicone is the most suitable support for performing the drying at 37 °C for 24 h as it allows an easy removal of the patch.

Through these studies, it was possible to select the most suitable gel composition, the support, and the most suitable drying conditions. However, the final object (patch) had some limitations, represented by the limited resistance during the removal from the printing support. Thus, the next studies had the aim of solving this problem.

The first strategy was to print multilayer patches. In particular, bilayer and three-layer patches were prepared using G4-βglu gel that worked using the previously selected parameters: 0.80 mm nozzle diameter, 100% infill, 40% flow, and 15 mm/s printing speed.

Comparing the dried bilayer and three-layer patches with the monolayer, it can be highlighted that in both cases, patch breaking occurred as a consequence of solvent removal by drying (Table 7).

From these results, it became apparent that this strategy is not suitable, and for this reason, it was decided to perform the extrusion of G4-βglu gel on a polymeric support that, after the post-printing drying process, should represent the backing layer of the final patch.

With this purpose, the polymeric support was prepared by casting method using a gel prepared using a biopolymer sodium alginate (Alg) that had the following composition: Alg 1.5% *w*/*w*, Gly 10% *w*/*w*, and water (W) 88.5% *w*/*w* optimized in a previous work [27]. Alg is a natural polysaccharide widely used in the health field for its excellent water solubility, biodegradability, biocompatibility, and no toxicity [36]. Moreover, it was successfully employed for patch preparation by solvent casting method [27].

**Figure 6:**
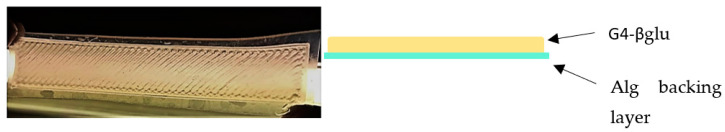
Image of the patch after drying (**left**) and schematic representation of the Alg_βglu A (**right**).

The final formulation, Alg_G4-βglu, was a bilayer patch consisting of the Alg backing layer on which a monolayer of G4-βglu gel was printed. After drying, the final object (Figure 6) is homogeneous, manageable, and resistant.

### 3.4. Patch Characterization

**Figure 7:**
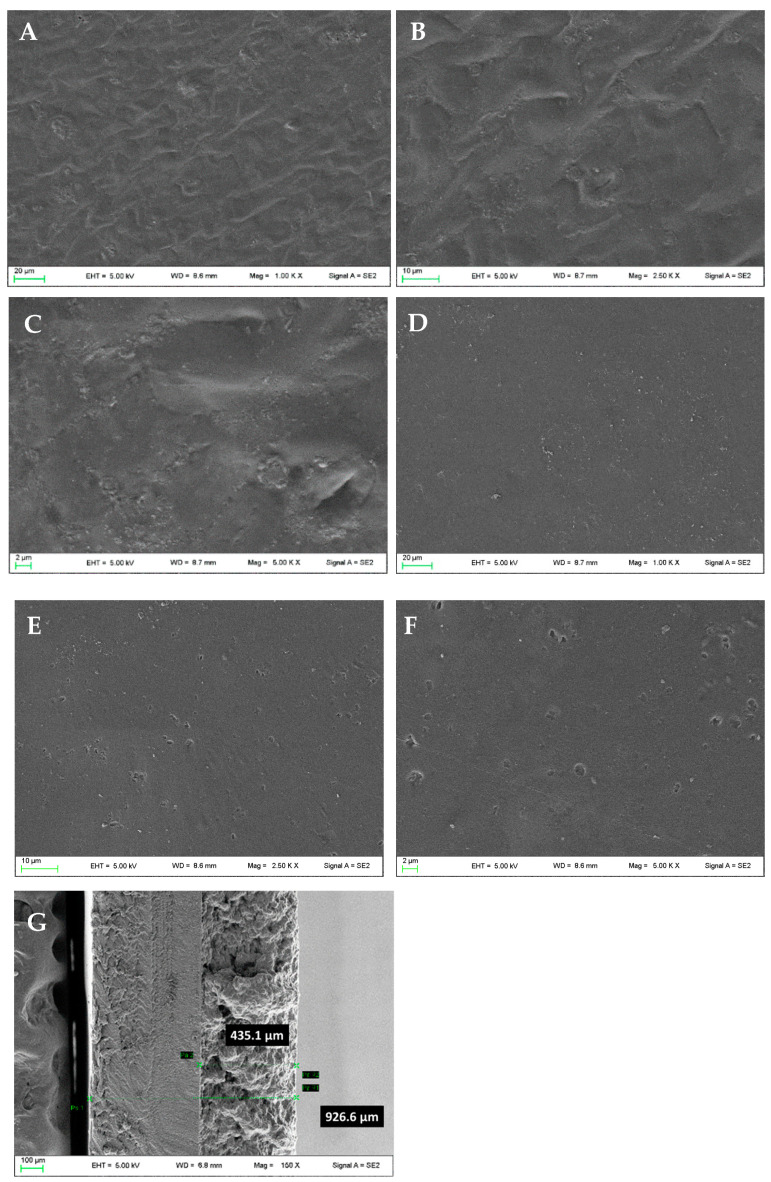
Micrographs of the patch: G4-βglu side (**A**–**C**), Alg side (**D**–**F**). Magnifications 1.00 KX (**A**,**D**), 2.50 KX (**B**,**E**), 5.00 KX (**C**,**F**), and thickness (**G**).

The prepared patch was deeply characterized. Firstly, the morphological analysis of both G4-βglu and Alg sides was performed by SEM. The G4-βglu side shows a wrinkled surface (Figure 7A–C), which is useful to obtain a better adhesion on the skin surface. The backing layer shows a smooth surface (Figure 7D–F). The measured thickness (Figure 7G) was 926 ± 18 µm, sufficiently thin to ensure imperceptibility after application and, at the same time, suitable to make the patch less susceptible to fractures.

#### 3.4.1. FT-IR

**Figure 8:**
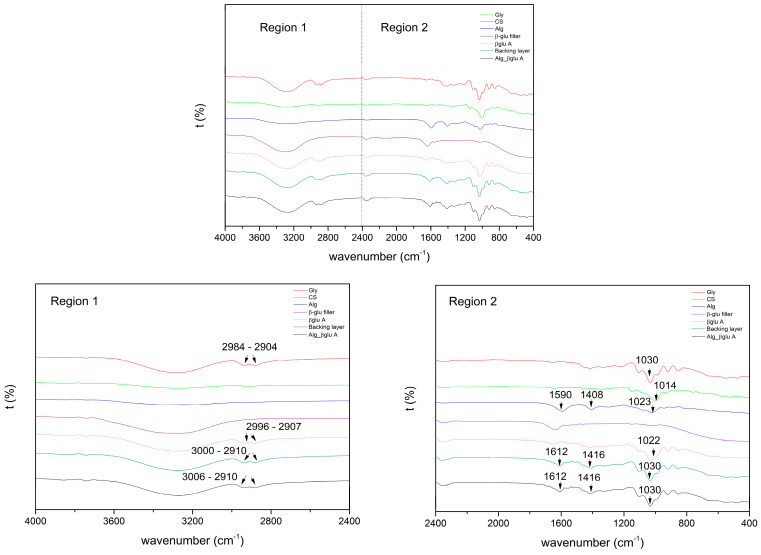
FT-IR spectra registered for βglu A patch, backing layer, and Alg_βglu A, as well as the βglu filler and the corresponding raw materials Gly, CS, and Alg.

In order to evaluate interactions between the two polymers CS and Alg, FT-IR spectra were registered (Figure 8).

FTIR spectrum of pure Gly shows five absorption bands located between 800 and 1150 cm^−1^ corresponding to the vibrations of the C-C and C-O bonds. Three broad bands at 850, 925, and 995 cm^−1^ correspond to the vibration of the C-C backbone; the peak at 1045 cm^−1^ is associated with the stretching of the C-O bond at C1 and C3, and the bond at 1117 cm^−1^ corresponds to the stretching of the C-O bond at C2. The effect of the interactions between Gly and distilled water can be analyzed by comparing the spectra of the CS powder with those of the βglu A patch, backing, and final patch containing different % Gly. In comparison with the IR spectra of the raw materials, the characteristic peaks of the saccharides were shifted (Figure 8). The peaks at 1014 and 1022 cm^−1^, for CS and Alg, respectively, could be assigned to C-O stretching and shifted to 1022 and 1030 cm^−1^, respectively [37]. Two characteristic absorption bands of pure Alg at 1590 and 1408 cm^−1^ for the asymmetric and symmetric stretching vibration of the –COO group, respectively, were shifted to 1612 and 1416 cm^−1^ in each patch containing Gly and Alg [36].

In addition, bands at 3006 and 2910 cm^−1^ were related to the formation of inter- and intramolecular bonding O-H groups between saccharides (CS and Alg) and Gly [38]. With these shifts, we can observe how the addition of Gly promotes hydrogen bonding interactions between CS and Gly and Alg and Gly [39].

#### 3.4.2. Mechanical Characterization

**Figure 9:**
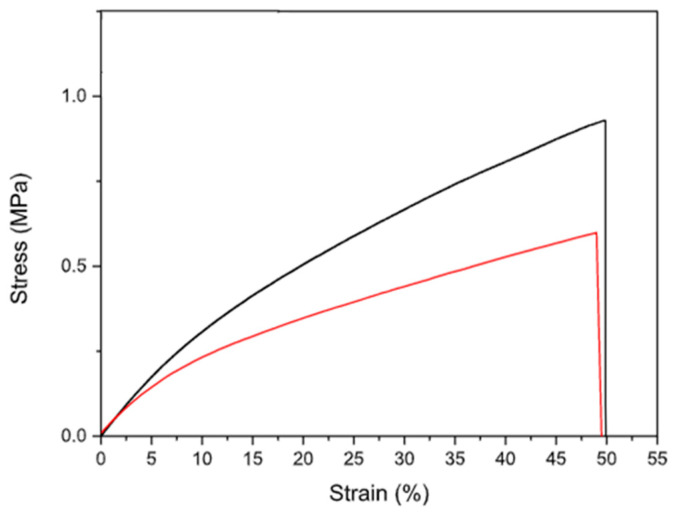
Tensile stress–strain curves for Alg_G4_βglu stored at R.T. for 1 week under vacuum (black line) condition and in oversaturated CaCl_2_ solution (red line).

The mechanical characterization of the final patch is essential as this formulation was designed to be applied on the skin and to conform to every type of surface; thus, it is necessary to know its elastic response. The mechanical properties of Alg_G4_βglu were evaluated after its storage at R.T. in two different humidity conditions: 1 week under vacuum condition and 1 week under saturated calcium chloride (CaCl_2_) solution (relative humidity R.H. 75%) [40]. The evaluation of mechanical performance after storage in different conditions is a crucial aspect needed to assess the stiffness of the polymer system prior to the application. It is necessary to specify that the mechanical parameters of hydrophilic polymers are strongly influenced by R.H., as the humidity acts as a plasticizer [41]. Moreover, patches intended to be applied on the skin must be resistant to mechanical solicitations to which they are subjected (e.g., during removal from packaging, application, and period of residence on the skin surface). Data in the literature suggest that the deformation requested for normal skin is between 61 and 70% [42]. The parameters measured for Alg_G4_βglu were elastic modulus (E), stress, and deformation at break (σb and εb) (Table 8). Figure 9 shows the stress–strain curves of Alg_G4_βglu stored in the two different R.H. conditions. In both cases, the presence of a backing layer realized using sodium alginate influences the mechanical response of the systems, as the alginate layer guarantees the integrity, strength, and uniformity of the systems. In fact, it was not possible to measure the mechanical properties of the monolayer G4_βglu due to its easy breaking during the removal from the silicone support. The presence of sodium alginate induces an improvement of tensile characteristics, contributing to a detectable increase in elastic modulus, strength, and deformation at break. An important contribution to the mechanical properties measured is due to the plasticizer. In fact, as observed by other authors, the use of both starch and alginate without a plasticizing agent gives a film with limited deformation, lower than 3% [43,44], whilst the presence of glycerol in the developed patch allows for the modulation of the humidity of the patch during the storage and before the application as it binds water molecules as observed from other authors [45]. The high amount of plasticizer used in the G4_βglu layer (29% *w/w*) and in the backing layer (10% *w/w*) greatly influences the mechanical response of the overall system. The storage in CaCl_2_ induces a slight reduction of mechanical performances with respect to the system storage under vacuum conditions (Table 8, Figure 9). This suggests the importance of suitable packaging in order to preserve the original features of the formulation.

#### 3.4.3. Ex Vivo Adhesion Studies

Ex vivo adhesion experiments were performed on healthy pig skin tissues using the system reported in Figure 2. For these studies, a circular patch of 12.56 cm^2^ (diameter of 4 cm), having the same shape and diameter as the bottom of the support (Figure 2C), was printed. The pig skin tissue was cut into circular pieces (diameter 4 cm) in order to obtain complete contact between the patch and tissue surfaces. The obtained data showed that the force necessary for patch detachment from skin is 0.77 ± 0.23 N. This value is mainly attributable to the hydrophilic character of the patch due to the main components of the formulation CS and βglu. It must be considered that the experiment was performed on intact skin in which the lipophilic character of the stratum corneum prevails. In the case of wounds, the patch should make contact with a more hydrophilic environment represented by the wound bed. Taking into account this aspect, it is reasonable to think that the in vivo application should produce a better result.

## 4. Conclusions

In the present study, a CS gel was developed with the following composition: CS 10% *w*/*w*; βglu 1% *w*/*w*; Gly 29% *w*/*w*; distilled water 60% *w*/*w*. This CS gel demonstrated suitable rheological properties for PAM-based 3D printing. In addition to optimizing the semisolid formulation, the following parameters for 3D printing were optimized: nozzle diameter 0.8 mm; layer height 0.2 mm; infill 100%; volumetric flow rate 3.02 mm^3^/s; print speed 15 mm/s.

To overcome the challenges related to the structural properties (low resistance during the removal from the printing support) of the final patches, several post-printing strategies were studied: (i) different drying temperatures, (ii) different drying times, and iii) different material supports for printing. The most successful condition was the drying at 37 °C for 24 h using silicone support. As patch separation from the silicone support is difficult, a further implementation was performed. CS gel printing was, in fact, performed on an alginate film support representing the backing layer of the final patch. Through this strategy, the physicochemical properties were considerably enhanced, obtaining a homogeneous and intact final product that can be used for the treatment of many skin problems.

It is important to underline that the patches were developed using a cheap and easy-to-use 3D PAM printer [45]. This technique represents an innovative tool useful in the field of personalized medicine, allowing for the obtaining of on-demand products able to meet the specific requirements of each patient [46,47]. Thanks to the CAD design, it is possible to prepare patches of different shapes and sizes, making the 3D printing PAM technique extremely versatile and allowing an ad hoc use for the patient, who will have an effective, manageable, and delicate treatment of wounds. On the other hand, the PAM technique allows for the development of biodegradable and biocompatible patches since it considers the environmental impact at each step of the process, from the choice of natural and sustainable raw materials without the use of synthetic materials (avoiding the problem of microplastics) to the development and final use of the printed formulations [48]. Moreover, it is an additive manufacturing process that does not generate waste [49], respecting humans, animals, and the environment, which meets the principles of the “One health” concept.

## Figures and Tables

**Table 1:** Parameters settings.

Parameters	Values	Units
Nozzle diameter	0.80–1.62	mm
Layer height	0.2–0.6	mm
Flow rate	30–80	%
Volumetric flow rate	1.5–48.2	mm^3^/s
Print speed	10–30	mm/s
Infill density	60–100	%

**Table 2:** Gels composition.

Gels	CS % (*w/w*)	Gly % (*w/w*)	Distilled Water % (*w/w*)
G1	10	60	30
G2	10	50	40
G3	10	40	50
G4	10	30	60
G5	10	20	70
G6	10	10	80

**Table 3:** Gels prepared using βglu as filler.

Gels	CS % (*w/w*)	Gly % (*w/w*)	Filler % (*w/w*)	Distilled Water % (*w/w*)
G3-βglu	10	39	1	50
G4-βglu	10	29	1	60
G5-βglu	10	19	1	70

**Table 4:** Printed patches at different percentages flow percentages.

Patch Code	Flow (%)	Volumetric Flow Rate (mm^3^/s)	Image	Evaluations
Patch F_30	30	2.26	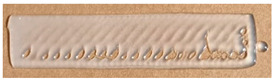	Incomplete extrusion
Patch F_40	40	3.02	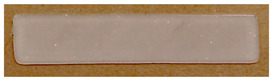	Complete extrusion, optimum layer thickness
Patch F_50	50	3.77	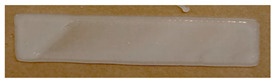	Rapid extrusion and non-continuous patch
Patch F_60	60	4.52	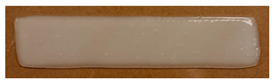	Over extrusion
Patch F_70	70	5.28	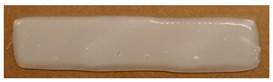	Very rapid extrusion, shape deformation
Patch F_80	80	6.03	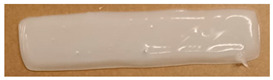	Very rapid extrusion with shape deformation and a high layer thickness

**Table 5:** Printed patches at different values of printing speed.

Patch Code	Speed (mm/s)	Image	Evaluations
Patch F_40_S_10	10	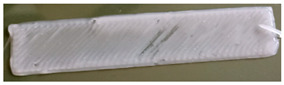	Slow printing
Patch F_40_S_15	15	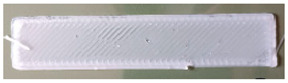	Optimum printingSmooth and continuous extrusion
Patch F_40_S_20	20	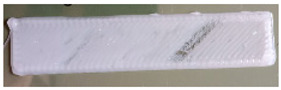	Rapid printing with low material extrusion
Patch F_40_S_25	25	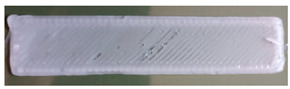	Very rapid printing
Patch F_40_S_30	30	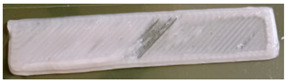	Very rapid printing, poor adhesion to the support

**Table 6:** Optimization of post-printing process (drying conditions and support).

Temperature/Time	Support	Results
70 °C/12 h [24]	PET	+
	PTFE	+
	silicone	+
40 °C/16 h	PET	+
	PTFE	+
	silicone	+
37 °C/24 h [35]	PET	++
	PTFE	++
	silicone	+++

The removal from the support was not easy (+), easy (++), very easy (+++).

**Table 7:** Image patches with one, two, and three layers.

Layer	Post Drying (βglu)
	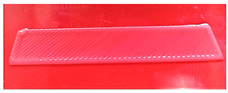
Monolayer	

	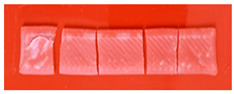
Bilayer	

Three layers	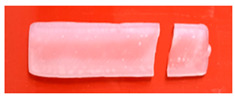

**Table 8:** Mechanical parameters measured for Alg_G4_βglu stored at R.T. for 1 week under vacuum conditions and in oversaturated CaCl_2_ solution.

Alg_G4_βglu	σ_b (MPa)_	ε_b (%)_	E (MPa)
under vacuum	0.92 ± 0.08	50 ± 1	3.80 ± 0.82
CaCl_2_	0.62 ± 0.07	48 ± 8	3.37 ± 0.31

## Data Availability

Not applicable.
